# Reduction in oral corticosteroid use in patients with severe allergic (IgE-mediated) asthma receiving omalizumab in a real-world setting

**DOI:** 10.1186/2045-7022-3-S1-P13

**Published:** 2013-05-03

**Authors:** Gert-Jan Braunstahl, Jan Chlumsky, Guy Peachey, Robert Maykut, Chien-Wei Chen

**Affiliations:** 1 St Franciscus Gasthuis, Department of Pulmonary Medicine, the Netherlands; 2 Thomayer Hospital, Charles University, Department of Pulmonary Disease, Czech Republic; 3 Novartis Pharmaceuticals UK Limited, Clinical Development, UK; 4 Novartis Pharma AG, Clinical Development, Switzerland; 5 Novartis Pharmaceuticals Corporation, Clinical Development, USA

## Background

Patients with severe allergic asthma (SAA) are often inadequately controlled despite available treatments including high-dose inhaled corticosteroids and long-acting β2-agonists. Use of oral corticosteroids (OCS) in SAA patients may not achieve full asthma control, and leads to significant long-term side effects. Omalizumab is a recombinant humanized monoclonal anti-immunoglobulin E (IgE) antibody approved in the European Union as an add-on therapy for patients with SAA. In clinical studies, omalizumab has been shown to reduce OCS use. Here we report the effect of omalizumab treatment on OCS maintenance use for up to 24 months in patients with SAA in the real-world eXpeRience registry.

## Methods

eXpeRience was a 2-year, multicentre, non-interventional, single-arm, observational registry initiated to collect data from patients receiving omalizumab for uncontrolled SAA. Data were collected on OCS maintenance use at baseline, Month 12, and Month 24. Parameters assessed were incidence of OCS maintenance use, total daily OCS dose and change from baseline, and time to reduction in OCS dose or stopping therapy.

## Results

At Month 24, 49% of the patients on OCS had discontinued their use and 20% had reduced their OCS dosage, this was incremental from Month 12. OCS maintenance use at baseline, Month 12 and Month 24 is summarized in Table 1.

**Table 1:** 

	Baseline N=916	12 months N=734	24 months N=643
Patients on OCS maintenance monotherapy, n (%)	262 (28.6)	118 (16.1)	91 (14.2)
Mean (SD) total daily OCS dose*, mg	15.49 (14.01)^a^	7.68 (10.94)^b^	5.77 (8.89)^c^
Mean (SD) reduction from baseline in total daily dose, mg	-	7.89 (13.77)^b^	9.95 (15.58)^c^
Patients with alteration in total OCS dose, n (%)			
**•** discontinuation	-	77 (40.7)^b^	82 (48.8)^c^
**•** reduction	-	31 (16.4)^b^	34 (20.2)^c^
**•** no change	-	76 (40.2)^b^	48 (28.6)^c^
**•** increased	-	5 (2.6)^b^	4 (2.4)^c^
Mean (SD) time to either reduction or discontinuation of OCS, days	-	198.5 (114.29)^d^	291.2 (210.86)^e^

* OCS dose was reported in prednisolone equivalent dose as mg per day. OCS **–** oral corticosteroid; SD **–** standard deviation; n **–** number of patients who received OCS at baseline and who provided OCS information at 12 months and 24 months (^a^n=246; ^b^n=189; ^c^n=168; ^d^n=108; ^e^n=116).

## Conclusion

Omalizumab reduced the need for maintenance OCS use in patients with severe allergic (IgE-mediated) asthma in a real-world setting. Reduction in OCS maintenance use may reflect better asthma control and decreases the risk of long-term morbidity of corticosteroid exposure.

